# Fast parallel construction of variable-length Markov chains

**DOI:** 10.1186/s12859-021-04387-y

**Published:** 2021-10-09

**Authors:** Joel Gustafsson, Peter Norberg, Jan R. Qvick-Wester, Alexander Schliep

**Affiliations:** 1 Institute of Biomedicine, Department of Infectious Diseases, University of Gothenburg, Gothenburg, Sweden; 2 Department of Computer Science and Engineering, University of Gothenburg — Chalmers University of Technology, Gothenburg, Sweden

**Keywords:** Variable-length Markov chain, Sequence analysis, Parallel algorithms, Alignment-free

## Abstract

**Background:**

Alignment-free methods are a popular approach for comparing biological sequences, including complete genomes. The methods range from probability distributions of sequence composition to first and higher-order Markov chains, where a *k*-th order Markov chain over DNA has $4^k$ formal parameters. To circumvent this exponential growth in parameters, variable-length Markov chains (VLMCs) have gained popularity for applications in molecular biology and other areas. VLMCs adapt the depth depending on sequence context and thus curtail excesses in the number of parameters. The scarcity of available fast, or even parallel software tools, prompted the development of a parallel implementation using lazy suffix trees and a hash-based alternative.

**Results:**

An extensive evaluation was performed on genomes ranging from 12Mbp to 22Gbp. Relevant learning parameters were chosen guided by the Bayesian Information Criterion (BIC) to avoid over-fitting. Our implementation greatly improves upon the state-of-the-art even in serial execution. It exhibits very good parallel scaling with speed-ups for long sequences close to the optimum indicated by Amdahl’s law of 3 for 4 threads and about 6 for 16 threads, respectively.

**Conclusions:**

Our parallel implementation released as open-source under the GPLv3 license provides a practically useful alternative to the state-of-the-art which allows the construction of VLMCs even for very large genomes significantly faster than previously possible. Additionally, our parameter selection based on BIC gives guidance to end-users comparing genomes.

## Background

Comparing biological sequences to identify phylogenetic or functional relations between species, or assigning DNA to a known species, is still one of the fundamental problems in biological sequence analysis. An optimal sequence alignment, or a best approximate match between two sequences, can be computed with dynamic programming in time proportional to the product of the sequence lengths [1]. As a response to the enormous growth in the number of DNA-sequences created by High-Throughput-Sequencing (HTS), advanced algorithms and data structures have been developed. These advancements greatly improve upon the complexity of the basic dynamic programming algorithm for specific tasks, such as DNA sequencing read alignment. For example, alignment heuristics employing the Burrows-Wheeler transform [2] allow mapping of gigabases of DNA against large genomes even on modest computing devices such as laptops. It is a testament to the growth of HTS data that even faster alignment-free approaches became a necessity for tasks such as analysis of RNAseq data [3].

Alignment-free approaches consider global aspects of sequences and have been a standard technique in bioinformatics from the beginnings, for a review see [4, 5]. For example, it was an early discovery that the GC-content is a discriminating variable when comparing genomes. Similarly, codon-usage was found to be species-specific [6].

Generalising these observations led to the modelling of global aspects of sequences, with simple statistics of sequence composition, from i.i.d. models to first and higher-order Markov chains. These models are generative and their parameters are estimated by counting words of length *k*, or *k*-mers [7]. Normalising probabilities over all *k*-mers with the same $(k-1)$-mer prefix, gives the probabilities $P(x_t | x_{t-k+1} x_{t-k+2} ... x_{t-1})$ of a *k*-th order Markov chain; the variables conditioned on are referred to as the context in the following. Statistics such as the $D_2$-statistic allow comparisons of different *k*-th order Markov chains [8] for applications such as clustering [9]. Clearly, *k* can not be chosen arbitrarily large as many *k*-mer counts will be zero, even for large genomes, as the number of *k*-mers grows exponentially in *k*.

Variable-length Markov chains (VLMCs), first introduced by Bühlman et al. [10], are a data-driven model class that include higher-order Markov chains, but do not prescribe one fixed context length. Instead, the context length varies depending on the amount of information the context provides, and how reliably probabilities can be estimated; see section “Support pruning” for details. Note, that this is in contrast to approaches where sets of *k*-mers without an underlying probability model are considered. These often focus on rare *k*-mers e.g. for species identification [11]—significantly similar in spirit to oligonucleotide probes in DNA-microarrays [12, 13]—or compute differences using $L_1$ [14] or Jaccard-distances [15].

The VLMC has been used in a wide variety of applications. Examples include identification of horizontal gene transfer [16], tracing plasmid origin [17], prediction of indel flanking regions in proteins [18], and as background for alignment-free sequence comparisons [19]. They form an alternative to *k*-mer set and Markov chain approaches. Note that they are also widely used outside bioinformatics [20, 21]. Prior work, no longer available [22], provided an efficient implementation based on lazy suffix trees [23], and a recent approach based on succinct data structures particularly focused on limited memory usage [24].

Our contribution consists of a practically fast, multi-threaded implementation of the lazy suffix tree approach and a hash-based, memory-efficient alternative. To the best of our knowledge, this is the first parallel implementation for learning VLMCs. We demonstrate scaling close to the theoretical maximum according to Amdahl’s law on the most relevant hardware, modern multi-core laptops and personal computers. This extends the scope of genomes which can be analysed in an alignment-free manner using VLMCs. To make the manuscript self-contained, we first present the lazy suffix tree idea. This is followed by a detailed discussion of our parallel implementation and an extensive validation demonstrating the computational efficiency.

## Implementation

The variable-length Markov chain of a sequence *S*, is composed of a set of *k*-mers *w*, with counts *N*(*w*). Those *k*-mers are connected through a probabilistic suffix tree. Here, the sequence *S* consists of the usual DNA alphabet $\Sigma :=\{A,C,G,T\}$, but other choices are possible. Each node in the probabilistic suffix tree corresponds to a *k*-mer $w\in \Sigma ^k$, $|w|=k$, and contains *N*(*w*), the conditional probabilities $p(\sigma | w)$ for every $\sigma \in \Sigma$, and references to children of the node. The children of *w* are the ($k+1$)-mers with *w* as a suffix. We will use *p*, *i*, *c* to refer to nodes in the tree, where *p* refers to a parent node of *i*, and *c* refers to a child node of *i*.

**Fig. 1:**
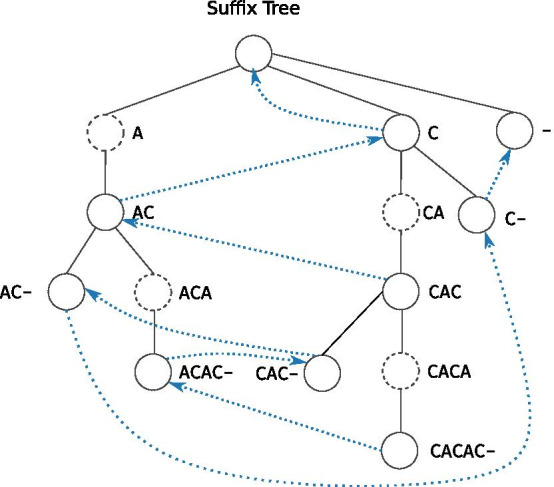
Illustration of the structure of a suffix and probabilistic suffix tree for the string CACAC. The ’-’ character is used to denote the end of the string. We illustrate implicit nodes (nodes with a single child, can be collapsed with a longer edge) as dashed circles. The dotted edges in the suffix tree illustrate the suffix links, which in the reverse direction are the edges of the probabilistic suffix tree. The full node label is included for clarity, and is not stored in each node in the tree

Our construction is based on an algorithm proposed by Schulz et al. [23], which uses a construction algorithm for lazy suffix trees [23, 25, 26]. Note, the original implementation of this idea is no longer available [22]. In the suffix tree, in contrast to the probabilistic suffix tree, child nodes have the parent as a prefix. However, it is possible to convert between the two trees via suffix links (see Fig. 1 for a comparison of the two tree types). For the reader’s benefit, we give a brief explanation of the suffix and lazy suffix tree, see the original articles [23, 25, 26] for full details. We then describe our parallelisation scheme and an alternative implementation with better performance.

### Suffix tree

A suffix tree is a data structure that sorts and stores a text’s suffixes in a tree, designed to, e.g., allow linear-time substring queries in a text. Each leaf node in the tree corresponds to a suffix, and every internal node corresponds to a shared prefix among suffixes. In addition, every internal node in the tree is branching, so that no node has a single child. Each edge in the tree is associated with a label, which when concatenated from the root to a leaf corresponds to the suffix of that leaf. This tree structure enables fast search queries since searches can be restrained to the relevant branches. A brief description of the suffix tree data structure from [25, 26] is provided below.

For every internal node *i* with parent *p*, the tree contains the edge label from *p* to *i* which enables reconstruction of *i*’s *k*-mer *w*, as well as a pointer to *i*’s child nodes. The *k*-mer is represented by its smallest start and end indices in the text, ${{\,\mathrm{{start}}\,}}(i)$ and ${{\,\mathrm{{end}}\,}}(i)$. This representation can be further optimised to store a single integer per node by utilising the longest common prefix ${{\,\mathrm{{lcp}}\,}}(i)$, the prefix shared by all nodes in *i*’s subtree, and, equivalently *w*. The length of the ${{\,\mathrm{{lcp}}\,}}(i)$ is denoted as $|{{\,\mathrm{{lcp}}\,}}(i)|=|w|$. The $|{{\,\mathrm{{lcp}}\,}}(i)|$ is used here as in [26] to maintain consistency with the construction algorithm given in the section “Lazy suffix tree”; in the modern literature this is typically called string depth. For each node, the tree contains the value ${{\,\mathrm{{node}}\,}}(i):={{\,\mathrm{{start}}\,}}(i)+|{{\,\mathrm{{lcp}}\,}}(p)|$, where *p* is the parent node of *i*. To find ${{\,\mathrm{{end}}\,}}(i)$, we locate the child *c* with smallest ${{\,\mathrm{{node}}\,}}(c)$, which gives the edge length between *p* and *i* as $|{{\,\mathrm{{edge}}\,}}(p,i)|:={{\,\mathrm{{node}}\,}}(c) - {{\,\mathrm{{node}}\,}}(i) = |{{\,\mathrm{{lcp}}\,}}(i)| - |{{\,\mathrm{{lcp}}\,}}(p)|$. This holds since the child *c* with smallest ${{\,\mathrm{{node}}\,}}(c)$ has the same start index as *i*, ${{\,\mathrm{{start}}\,}}(c) = {{\,\mathrm{{start}}\,}}(i)$. This gives the end index of *i* as ${{\,\mathrm{{end}}\,}}(i)={{\,\mathrm{{node}}\,}}(i) + |{{\,\mathrm{{edge}}\,}}(p,i)|$ and the longest common prefix length of *i* as $|{{\,\mathrm{{lcp}}\,}}(i)| = |{{\,\mathrm{{lcp}}\,}}(p)| + |{{\,\mathrm{{edge}}\,}}(p, i)|$. The label of each edge can then be found with the indices ${{\,\mathrm{{node}}\,}}(i)$ and ${{\,\mathrm{{end}}\,}}(i)$ into the text. To recover ${{\,\mathrm{{start}}\,}}(i)$, it is sufficient to know the value of ${{\,\mathrm{{node}}\,}}(i)$, and the $|{{\,\mathrm{{lcp}}\,}}(p)|$, which needs to be kept in memory or reconstructed during iteration by finding $|{{\,\mathrm{{edge}}\,}}(p, i)|$ as described above. For leaf nodes *l*, storing only ${{\,\mathrm{{start}}\,}}(l)$ is sufficient, as the end index is always the end of the text. The root *r* of the tree corresponds to the empty string and has the values ${{\,\mathrm{{node}}\,}}(r)=0$ and $|{{\,\mathrm{{lcp}}\,}}(r)|=0$.

To efficiently find child nodes, all nodes are stored in a one-dimensional vector called *table* with children of the same node adjacent to each other. This allows each internal node *i* to store only the index of its first child, ${{\,\mathrm{{first\_child}}\,}}(i)$, in the *table*. One extra byte of memory is used to keep track of the last child, stored in a vector called *flags*. In the same byte, by using byte flags, nodes are also labeled as leaves. In conclusion, the data structure uses two integers and one byte for each node *i*, the values ${{\,\mathrm{{node}}\,}}(i)$ and ${{\,\mathrm{{first\_child}}\,}}(i)$ which are stored in the *table* vector, and the byte flag in the *flags* vector.

### Lazy suffix tree

Constructing the entire suffix tree is not needed for the variable-length Markov chain, as only a subset of *k*-mers is of interest. Therefore, we can avoid unnecessary work by lazily computing a subtree of the suffix tree. The lazy suffix tree [25, 26] thus delays node construction until explicitly needed. For instance, if an application only needs suffixes starting with ’A’, it can avoid computing suffixes starting with ’C’, ’G’ or ’T’.

The lazy suffix tree data structure is similar to the suffix tree described previously but also supports nodes that have not been evaluated. Specifically, each unevaluated node has to keep track of the suffixes in the node’s subtree. To this end, an additional array called *suffixes* is created that contains the start index of each suffix. Each unevaluated node *i* is assigned a contiguous range $[{{\,\mathrm{{left}}\,}}(i), {{\,\mathrm{{right}}\,}}(i)]$ in the *suffixes* array, containing all suffixes with node *i*’s *k*-mer as common prefix. The boundary indices ${{\,\mathrm{{left}}\,}}(i)$ and ${{\,\mathrm{{right}}\,}}(i)$ of an unevaluated node *i* of this range in *suffixes* is stored in *table*, in the place of ${{\,\mathrm{{node}}\,}}(i)$ and ${{\,\mathrm{{first\_child}}\,}}(i)$, and the unevaluated state is stored in the *flags* vector.

Evaluating a node *i* requires four steps. First, the algorithm calculates the longest common prefix of the suffixes in $[{{\,\mathrm{{left}}\,}}(i), {{\,\mathrm{{right}}\,}}(i)]$. Second, the suffixes are lexicographically sorted with a counting sort (e.g. [27]) by their first character after the common prefix, and by suffix length. This puts the longest suffix starting with ’A’ (assuming it exists) first in the range and gives a subrange per character. Third, new children are created. For new unevaluated nodes *c*, the ${{\,\mathrm{{left}}\,}}(c)$ and ${{\,\mathrm{{right}}\,}}(c)$ of their subrange in *suffixes* for the corresponding character is appended to the *table*. For leaf nodes *c*, the start of the suffix ${{\,\mathrm{{start}}\,}}(c)$ is appended. Fourth, we set node *i*’s values in *table* to ${{\,\mathrm{{node}}\,}}(i) = {{\,\mathrm{{start}}\,}}(i) + |{{\,\mathrm{{lcp}}\,}}(p)|$, with *p* as the parent of *i*, and ${{\,\mathrm{{first\_child}}\,}}(i)$, the index of *i*’s first child.

Initially, the root node *r* is assigned ${{\,\mathrm{{left}}\,}}(r)=0$ and ${{\,\mathrm{{right}}\,}}(r)=|S|-1$. The evaluation of the root node is almost equivalent to every other node, except for ${{\,\mathrm{{node}}\,}}(r)$ getting a value of 0.

Note that the algorithm only creates branching nodes. Specifically, only branching nodes are created since step one above will skip all non-branching (implicit) nodes as a non-branching node will not correspond to a longest common prefix among the suffixes in $[{{\,\mathrm{{left}}\,}}(i),{{\,\mathrm{{right}}\,}}(i)]$.

The expected time complexity of the algorithm is $\mathcal {O}(n \log n)$ with *n* as the length of the sequence [25]. In the worst case it can reach $\mathcal {O}(n^2)$ [25]. However, due to the lazy evaluation, it is still fast in practice.

### Suffix links

To convert the suffix tree into a probabilistic suffix tree, we use suffix links. These correspond to edges going from *uv* to *v*, where $u \in \Sigma$ and $v \in \Sigma ^*$. This is the reverse direction compared to the parent edges in the suffix tree. We use the suffix link reconstruction algorithm proposed by Maaß [28].

The intuition behind the algorithm is that for a suffix *i*, with starting index ${{\,\mathrm{{suf}}\,}}(i)$, the suffix link of *i* points to the suffix *j* with ${{\,\mathrm{{suf}}\,}}(j) = {{\,\mathrm{{suf}}\,}}(i) + 1$. This next suffix *j* will be one shorter and lack the first character. Therefore, if we can find the suffix with that next index, we can find the suffix links. Note that for leaf nodes/suffixes *i*, ${{\,\mathrm{{suf}}\,}}(i)={{\,\mathrm{{start}}\,}}(i)$, the extra definition is included to highlight that ${{\,\mathrm{{suf}}\,}}(i)$ is only defined for suffixes.

The algorithm iterates the tree twice. First, in the prepare step, for a node *n* with potential children *c* the algorithm performs the following recursive steps. If *n* is a suffix, the value of ${{\,\mathrm{{suf}}\,}}(n)$ is returned. Instead, if *n* is a branching node, the algorithm makes a recursive call to each *c*, which returns the smallest ${{\,\mathrm{{suf}}\,}}(j)$ among the suffixes *j* of *c*. From these smallest ${{\,\mathrm{{suf}}\,}}(j)$ from each *c*’s subtree, the second smallest is selected, ${{\,\mathrm{{cause}}\,}}(n)=\text {min2}_{c \in {{\,\mathrm{{children}}\,}}(n)} \min _{j \in {{\,\mathrm{{suffixes}}\,}}(c)} {{\,\mathrm{{suf}}\,}}(j)$. Here, $\text {min2}$ refers to the function that returns the second smallest value. The second smallest value of each child is selected to ensure that each ${{\,\mathrm{{cause}}\,}}(n)$ is unique. The uniqueness follows from each ${{\,\mathrm{{suf}}\,}}(j)$ being unique, and that only the smallest ${{\,\mathrm{{suf}}\,}}(j)$ value from each child is used, while the second smallest is never propagated upwards in the tree. The value ${{\,\mathrm{{cause}}\,}}(n) + 1$ is used as index to store the node *n*, and the node’s string depth *d*, which is equivalent to the $|{{\,\mathrm{{lcp}}\,}}(n)|$, in an array. Finally, the *very* smallest of all ${{\,\mathrm{{suf}}\,}}(j)$ among the suffixes *j* of every *c* is returned.

During the second iteration, which is the compute step, the algorithm assigns the suffix links. The tree is traversed depth-first, the latest branching node at each string depth is recorded in a vector, and when the algorithm encounters a suffix *i* with a ${{\,\mathrm{{suf}}\,}}(i)$ value that was set during the first iteration, a suffix link can be established. The node *n*, with depth *d*, stored at ${{\,\mathrm{{suf}}\,}}(i)$ is assigned a suffix link pointing to the parent of *i* at depth $d - 1$, which is guaranteed to exist since this is a suffix link. The correctness of this follows the intuition given before. Since the suffix link of a suffix *j* with ${{\,\mathrm{{suf}}\,}}(j)={{\,\mathrm{{cause}}\,}}(n)$ will point to *i* as ${{\,\mathrm{{suf}}\,}}(i)={{\,\mathrm{{cause}}\,}}(n)+1$, the suffix link from *n* at string depth *d*, which is a parent of *j*, must point to the parent of *i* at string depth $d - 1$. Note also that every ${{\,\mathrm{{cause}}\,}}(n) + 1$ value from the first iteration will be encountered during the second iteration as every such value will correspond to a unique suffix in the subtree of the destination of *n*’s suffix link. We devise a similar approach to determine the suffix links for the leaves.

Since the lazy suffix tree includes unevaluated nodes, but the suffix link reconstruction by Maaß [28] is designed only for the standard suffix tree, a slight modification to the algorithm has been introduced. During the prepare step, when an unevaluated node *i* is encountered, the smallest suffix in the subtree of *i* is determined and propagated up the tree in the recursion. During the compute step, all suffixes *c* in the subtree of the unevaluated node *i* are iterated to search for previously stored ${{\,\mathrm{{suf}}\,}}(c)$ values. However, due to the symmetric nature of how the probabilistic suffix tree will be built, this last step is not necessary as all required suffixes will be reached regardless.

The time complexity of the suffix link reconstruction is $\mathcal {O}(N)$ with *N* as the number of nodes in the tree, which is at most $\mathcal {O}(n)$ with *n* as the sequence length [28]. With the same argument, the memory complexity is $\mathcal {O}(n)$ [28].

### Probabilistic suffix tree

**Fig. 2:**
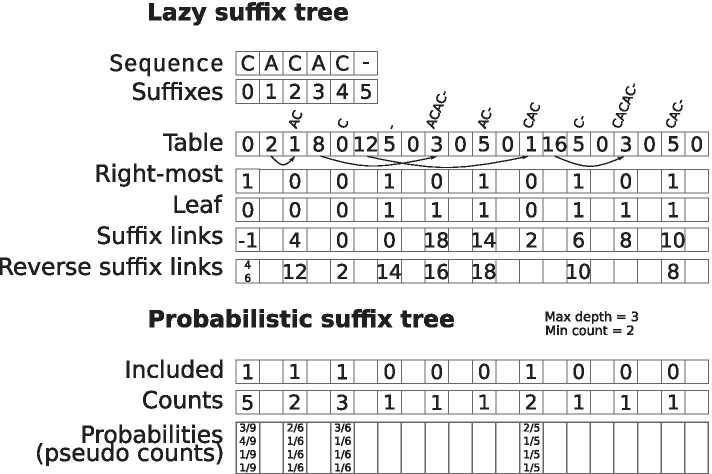
Illustration of the data structures in the variable-length Markov chain for the sequence CACAC. The ’-’ character is used to denote the end of the sequence. Above each node in *table*, we illustrate the node label. The *Right-most* and *Leaf* vectors are in practice stored in a single vector with byte-flags, which also marks nodes as unevaluated. The reverse suffix links defines the edges of the probabilistic suffix tree. We do not include implicit nodes here to make the example less verbose

With the lazy suffix tree and the suffix links, we can build the probabilistic suffix tree, which represents the variable-length Markov chain. The tree uses the reverse of the suffix links (the Weiner links) as edges, and the suffix tree’s edges for next-symbol probabilities. We follow Schulz et al. [23] for constructing the probabilistic suffix tree, thus building the tree in two stages: support pruning and similarity pruning. Between these two stages, the suffix links are constructed. Figure 2 provides an overview of the data structures needed for the tree.

#### Support pruning

The support pruning phase builds the lazy suffix tree by including every *k*-mer *w* with $|w|=k$ and count *N*(*w*) that fulfils Eq. (1), namely those that occur at least *t* times and are at most *L* long,

1 $$\begin{aligned} N(w) \ge t \quad \text {and} \quad |w| \le L. \end{aligned}$$

#### Similarity pruning

The similarity pruning phase removes nodes with similar probability estimates to their parents (Eq. (3)). This removes redundant nodes from the tree and is a crucial distinction between a Markov chain and a variable-length Markov chain. The next-symbol probabilities $\hat{p}(\sigma | w), \, \sigma \in \Sigma$ of each node are estimated as

2 $$\begin{aligned} \hat{p}(\sigma | w) = \frac{N(w\sigma )}{\sum _{c \in \Sigma } N(wc)}. \end{aligned}$$

Furthermore, we use pseudo-counts, meaning that every *N*(*w*) is increased by one to avoid estimated probabilities of 0. With $c\in \Sigma$, and $w\in \Sigma ^{k-1}$, nodes with *k*-mer *cw* are pruned by their level of similarity to their parent *w* in the probabilistic suffix tree, based on the Kullback-Liebler divergence [29],

3 $$\begin{aligned} \Delta _{cw} = N(cw) \sum _{\sigma \in \Sigma } \hat{p}(\sigma | cw) \log \frac{\hat{p}(\sigma |cw)}{\hat{p}(\sigma | w)} < K. \end{aligned}$$

Where *K* is specified by the user. Other options for similarity-pruning than the Kullback-Leibler divergence are possible (e.g. [30–34]), but is not the focus of this work. However, they should be easy to implement in our parallel framework.

### Implicit nodes

Edges can have arbitrary lengths in the suffix tree, as they can be labelled with multiple characters, and the nodes that are thus excluded are referred to as implicit nodes. However, the variable-length Markov chain requires edges of length one to explicitly represent each node. Explicitly representing each node is necessary to include nodes that would otherwise be cut off by the max depth *L*, but also allows for pruning parts of implicit nodes. Therefore, we modify the lazy suffix tree to expand these implicit nodes. This requires a slight modification to how leaves are stored. Since leaves otherwise only require a single integer, but can implicitly contain other nodes, we modify the leaf representation to two integers.

We add implicit nodes as any other node in the suffix tree. Specifically, the first child index is adjusted to a newly added node, and the last such added node contains the index to the previous child. The ${{\,\mathrm{{node}}\,}}(i)$ value for each node *i* is adjusted so that the $|{{\,\mathrm{{edge}}\,}}(p, i)|$ is one for every node.

The implicit nodes also require suffix links. After computing these for all explicit nodes, these suffix links can be found by checking the suffix link of an implicit node’s parent. For every implicit node *i* with parent *p*, *p*’s suffix link destination *j* is computed. One of the children of *j* has to be the suffix link of *i*, and to determine which one we check their corresponding *k*-mer’s last character. Every other character will be correct due to the relationship between the two nodes.

### Parallelisation of variable-length Markov chain construction

**Fig. 3:**
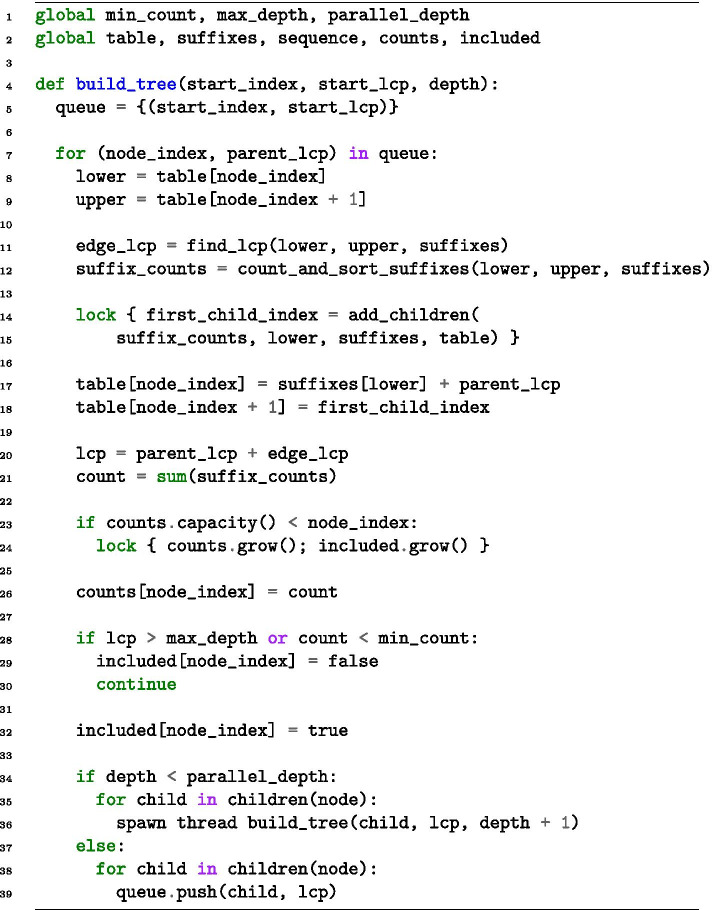
Pseudo-code for the parallel construction of the suffix tree. Nodes are evaluated as described in the “Lazy suffix tree” section. The *add_children* function adds children to the *table* vector. We use the *lock* keyword to illustrate where synchronisation locks are needed in the algorithm. We have excluded implicit nodes and leaf nodes to reduce verbosity

Our main contribution is the parallelisation of the algorithm, described in further detail in a thesis report [35]. We focus our parallelisation efforts on the support pruning and suffix link construction. By profiling the sequential algorithm, we find that these stages take approximately $92\%$ of the algorithm’s runtime. The other parts include input, output, and similarity pruning, and are excluded from parallelisation. We provide pseudo-code of the parallel support pruning in Fig. 3.

The design of the lazy suffix tree lends itself nicely to parallelisation. Specifically, each unevaluated node *i* is assigned an exclusive subrange $[{{\,\mathrm{{left}}\,}}(i),{{\,\mathrm{{right}}\,}}(i)]$ in the *suffixes* array, and each node evaluation consists mainly of iterating the suffixes in the subrange. Almost every part of the evaluation of nodes is independent, except for adding new children. When new nodes are added, they are appended to the *table* vector, where it is crucial that the children immediately follow each other, which thus requires exclusive access to the *table*. Afterwards, the resulting *k*-mer is checked to see if it satisfies Eq. (1), which can also be done in parallel. If a node does not satisfy Eq. (1), it does not need to be added to the *table*.

The lazy suffix tree algorithm does not support removing nodes. Therefore, the variable-length Markov chain requires an additional vector to mark nodes as included or excluded. Furthermore, the count *N*(*w*) of each *k*-mer *w* is stored upon node evaluation. The counts can be computed later, by iterating and counting the suffixes in the subtree of a node, but we have found that storing the counts saves a significant amount of computation time, but at the cost of some memory.

To resolve the exclusive access to the vectors, our approach requires two synchronisation locks for the support pruning phase. The first lock is acquired for every node evaluation, when nodes are added to the *table* vector. When each node contains a lot of suffixes, this allows for parallel execution of a large part of the work. However, as the depth of the tree increases and the number of suffixes for each node decreases, this synchronisation lock significantly impacts the parallelisation potential. The other lock is acquired when the vector containing node counts is resized, which does not occur on every node evaluation, and has less of an impact on the parallel performance.

As we build the tree top-down, the granularity of the parallel tasks is controlled by creating new threads for the first few branching nodes. Each thread becomes responsible for evaluating all nodes in its subtree. To maximally use the available cores, we create new threads for every node at every depth below a user-specified value. The first few nodes in the tree typically have the largest workloads. Thus, this recursive spawning of new threads is crucial for the speedup of the algorithm. The root node expansion, which is the most expensive node evaluation, can be parallelised using other techniques. However, since it takes a relatively small amount of runtime, this has not been implemented here.

**Fig. 4:**
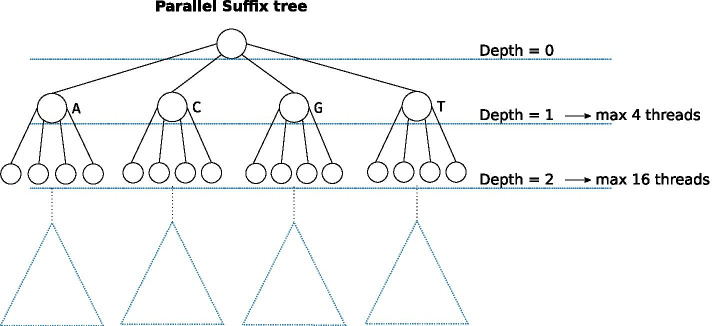
Parallel execution of the construction or iteration of the tree. At every node depth, new threads are spawned up until a *parallel_depth* threshold

The number of threads is specified with the user-controlled parameter *parallel_depth*, which specifies for how many levels of the tree we spawn new threads (see Fig. 4). For example, setting the *parallel_depth* to 1 with the DNA alphabet creates 4 threads, while a value of 2 creates 4+16 threads, one each for the initial 4 nodes which in turn spawn 4 threads each.

This granularity scheme works best when the average occurrence of every character in the alphabet is roughly equal. However, it is common for genomic sequences to be biased, with differing amounts of GC-content, which gives some threads more work than others. Therefore, to increase CPU utilisation, it might be useful to specify a *parallel_depth* for more threads than are available on the system.

An alternative to this granularity scheme is to use a worker pool, where each tread would be assigned arbitrary nodes to evaluate. This could have mitigated the effect from the imbalance of nucleotide content and allowed the parallelisation to scale to an exact user-specified amount of threads. However, our approach, in theory, makes it easy to execute parts of the algorithm on a different machine, as a limited amount of data has to be copied, specifically, while the full text has to be copied, the *suffixes* and *table* vectors could be local to each machine. This requires some more work, and the results from the different machines would need to be merged. Furthermore, our granularity approach allows for a slightly better cache-locality as each thread works on a subset of the suffixes. The main source of cache-misses in the algorithm, however, comes from the random distribution of pointers in the *suffixes* array, thus, the better cache-locality of this approach will have a minor impact overall.

Instead of all threads sharing the *table* vector, an alternative approach could have had each thread working on a local *table* vector. This approach was explored, and while it decreases the need for locks, the subsequent merge step to join the *table* vectors and update the indexes and next-child pointers makes the approach slower in practice.

### Parallelisation of suffix link construction

Most of the work per node in the suffix link construction is independent of other nodes. Specifically, there are no simultaneous accesses to the same memory, and all of the data structures can be pre-allocated, so there is no need for synchronisation primitives. However, in contrast to the suffix tree construction, the iteration order is important for the assignment of suffix links. During the prepare step, we propagate results from children to parents with shared memory between the parents and their child evaluations. During the compute step, the algorithm needs to keep track of the current node’s parents, up to the root node. These parents are stored in an array with an entry per depth in the tree. Each thread copies the array of parents to every new thread it spawns. This is the only part of the parallel implementation that requires extra memory. The depth of the tree is constant as the user provides it as the maximum *k*-mer length, so the copied memory is small.

We apply the same granularity approach to the suffix link construction as in the suffix tree construction. Each subtree rooted at the first nodes is assigned a separate thread, which computes the corresponding subtree’s suffix links.

### Hash map implementation

**Fig. 5:**
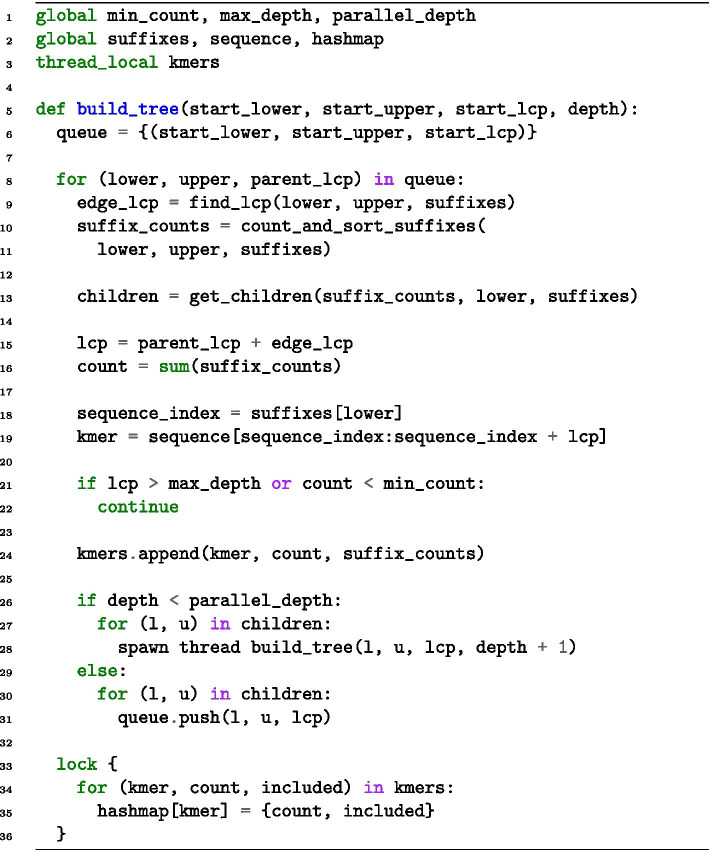
Pseudo-code for the parallel hash-map construction of the suffix tree. We use the *lock* keyword to illustrate synchronisation locks. The get_children function calculates the bounds of each child in the array *suffixes*. We have excluded the details regarding leaves and implicit nodes. Leaves do not need evaluation, so can be added to *kmers* as they are found. For implicit nodes, we add one node per *edge_lcp*

The parallelisation we have discussed so far requires two synchronisation locks. This is not an issue at the start of the construction when sorting and counting suffixes takes some time, but the locks drastically decrease the parallelisation potential for deeper levels of the tree. Therefore, we consider an alternative algorithm where the shared vector is removed, and *k*-mers are stored in a hash map instead of a tree. This approach will also allow us to skip the suffix link construction, which requires a significant amount of time and memory. We illustrate the algorithm with pseudo-code in Fig. 5.

This alternative algorithm uses a modified version of the lazy suffix tree, to only count *k*-mers. Since everything but adding new children to the tree is independent, the goal is to remove the vector that represents the tree structure. Therefore, this algorithm will only be useful if the tree is iterated once, and the result stored elsewhere.

We remove the dependence on the tree structure by modifying the iteration process surrounding the tree construction. Any breadth-first iteration of the lazy suffix tree requires a queue *Q* to keep track of the nodes that will be iterated. In the original algorithm, *Q* contains each node’s index *i* and the $|{{\,\mathrm{{lcp}}\,}}(p)|$, where *p* is the parent of *i*. Here, *i* is replaced with the indices ${{\,\mathrm{{left}}\,}}(i)$ and ${{\,\mathrm{{right}}\,}}(i)$ into the *suffixes* array. These two indices are the only requirement to evaluate a node *i* in the lazy suffix tree, and thus, the *table* vector can safely be removed. Having evaluated a node, its corresponding *k*-mer *w*, occurrence count *N*(*w*), and next-symbol counts are known, which are stored in a hash map, although other options are possible. Depending on whether *w* and *N*(*w*) satisfies eq. (1), the node’s children *c* are added to the queue *Q* with their corresponding indices ${{\,\mathrm{{left}}\,}}(c),{{\,\mathrm{{right}}\,}}(c)$ into *suffixes*, or if they are leaves, the index ${{\,\mathrm{{start}}\,}}(i)$ into the sequence. Implicit nodes from the suffix tree are expanded as expected.

For the parallelisation of this alternative algorithm, we do not store the *k*-mers in a shared hash map. Instead, they are temporarily stored in a vector per thread. This allows each thread to fully evaluate its respective subtree without any synchronisation primitives. After the iteration of the tree has finished, all *k*-mers *w* with *N*(*w*) and probabilities $p(\sigma |w)$ are added to a hash map. When all threads finish simultaneously, this causes some threads to have to wait to access the hash map before they can store their results.

This parallelisation approach requires some extra memory for the thread-local vectors. These vectors store the *k*-mer *w*, count *N*(*w*), and next-symbol counts. Thus, in contrast to the original algorithm, there is some memory overhead. However, since the data is later moved to a shared hash map, it does not have a large impact on the algorithm’s maximum memory usage.

We use the same granularity scheme as for the lazy suffix tree, where each subtree up to a given depth is assigned a thread. The temporary arrays where the threads store *k*-mers during the iteration are local to each thread and do not require synchronisation until after the thread has finished iteration of its subtree. Furthermore, since all children of the suffix tree and probabilistic suffix tree can be found by hash-lookup of the suffix or prefix of *w* respectively, we don’t need to compute suffix links. This results in a significant improvement in both computation time and memory usage.

### Model selection

The present algorithm has three parameters, the min count (*t* in eq. (1)), the max depth (*L* in eq. (1)) and the Kullback-Leibler threshold (*K* in eq. (3)). To select appropriate value of these parameters, while avoiding over-fitting the data, we find optimal parameter settings for each sequence with the Bayesian information criterion (BIC) [36].

Model selection using BIC is well established for variable-length Markov chains [37–39]. Mächler and Bühlmann [37] assert that model selection with BIC is the best choice for long sequences based on the theoretical guarantees provided by the BIC. In subsequent work, BIC was found to be a consistent estimator for the variable-length Markov chain [38, 39].

The BIC is based on the likelihood $P_M(S)$ of the training sequence *S* with length |*S*| under the model *M* and penalises the number of free parameters $\text {card}(M)$ in the model: $\text {BIC} := \text {card}(M) \log {|S|} - 2 \log P_M(S)$. The free parameters of the model are based on the leaves of the probabilistic suffix tree $\mathcal {L}(M)$, which are the *k*-mers without at least one child in the tree. Specifically, $\text {card}(M) := (|\Sigma | - 1)\mathcal {L}(M)$, as the free parameters correspond to the probabilities of the model. The free parameters include only the leaves of the tree since all internal nodes can be extended to a more specific context and thus are redundant. This is the same definition as has been used for model selection of variable-length Markov chains previously [37]. The parameter settings with the lowest BIC score are referred to as the optimal parameters.

### Dataset

We analyzed the performance of the parallelised algorithms on genomes from 12 Mbp to 22Gbp size, see Table 1, selected from a range of taxonomic classes. This range of sequence sizes includes almost all sequenced genomes to date, but due to the memory constraints of our test machine excludes the 6 largest currently sequenced genomes available at NCBI in 2021-06. We retrieved the genomes from GenBank’s FTP server and trained one variable-length Markov chain per genome.

**Table 1:** The genomes used to benchmark the performance of the algorithms

Organism	GenBank identifier	Sequence length (bp)	GC %
*Pandoravirus salinus*	NC_022098.1	2,473,870	61.72
*Sorangium cellulosum*	GCF_004135735.1	11,261,481	72.58
*Drosophila melanogaster* (Fruit fly)	GCA_004798055.1	133,403,897	42.12
*Oryza sativa* (Rice)	GCA_001623365.2	387,424,359	43.61
*Symbiodinium kawagutii* (Dinoflagellate)	GCA_009767595.1	935,067,369	45.54
*Homo sapiens* (Human)	GCA_000001405.28	3,099,706,404	41.04
*Palaemon carinicauda* (Crustacean)	GCA_004011675.1	6,699,723,695	37.37
*Pinus taeda* (Loblolly Pine)	GCA_000404065.3	22,103,635,615	37.45

The sequences are selected to represent various domains, with viruses, bacteria, insects, plants and animals represented, and with an emphasis on sequence length

### Availability and implementation

Our algorithm is implemented in C++ and uses the SeqAn3 library [40], and the hash-map implementation from [41]. An open-source implementation of the method is made available at https://github.com/Schlieplab/PstClassifierSeqan. The data structures are provided as a header-only library for easy inclusion in other projects. We also provide command-line applications to train single or multiple variable-length Markov chains and score other sequences with the negative log-likelihood, and a small Python interface for training and scoring sequences.

## Results

We compare the variable-length Markov chain implementations based on the lazy suffix tree to implementations by Cunial et al. [24], Lin et al. [42], Dalevi et al. [16], and Bejerano [43]. We refer to the algorithms by the name of their first author, and our implementations as Tree and HashMap respectively. The Cunial algorithm uses the Burrows-Wheeler transform to create a memory-efficient index, while the Lin algorithm is based on the suffix-array, and the Bejerano algorithm is based on the suffix tree. Unfortunately, since the paper introducing the Dalevi implementation focuses on the statistics, they don’t explain their realisation. Ours is the only parallel implementation, although parallelisation of the underlying data structures of the other implementations is possible. The lazy suffix tree construction has previously been demonstrated to be faster than both the Bejerano and a suffix array version [23], although the implementation is no longer available [22]. Furthermore, the Lin algorithm, based on the suffix array, has previously been shown to be three times faster in constructing the variable-length Markov chain than the Cunial algorithm [24].

The benchmarking was performed in a singularity container on a Debian 10.7 Linux cluster with Intel$^{\textregistered }$ Xeon$^{\textregistered }$ Gold 6130 CPUs (16 cores, 2.1GHz, with two CPUs per compute node) and 384 GiB memory. All codes are compiled with GCC 8.3.0 and the ’-03’ and ’-march=native’ compiler options, which are the default in Cunial. We measure peak memory usage of the construction and scoring steps with the heaptrack program, and the running time as the wall clock CPU time, including input and output.

Due to the excessive memory requirements, up to 1000 times more memory than the others, we exclude Dalevi from further analysis. Similarly, perhaps due to indexing with 32-bit integers, the Lin implementation only computes results for the two smallest test genomes. On the smallest genome, it matches, respectively is 4 times slower, than sequential HashMap for a min count of 10 and 100. The Bejerano is at least 100 times slower than both the HashMap and Tree even on the smallest genomes, while being less memory efficient than the Cunial algorithm. Due to these issues, we exclude the Dalevi, Lin and Bejerano algorithms from further benchmarks.

**Fig. 6:**
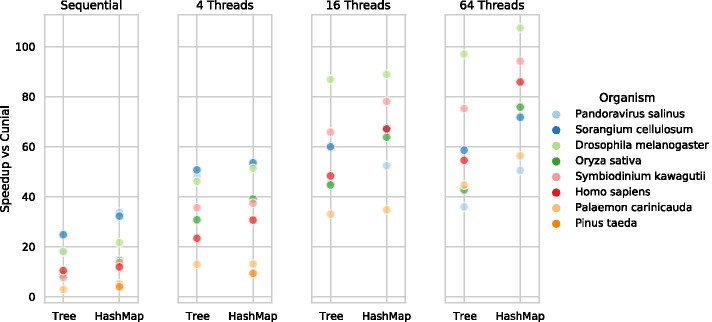
Speedup for the Tree and HashMap algorithms compared to Cunial. With parallellisation, the Tree and HashMap are up to 91 and 107 times faster than Cunial, respectively. Results for *Pinus taeda* are missing in some cases due to insufficient memory on the benchmarking machine, see text for details

Model selection using BIC was run on an extended set of genomes of varying sizes with a grid search of min count (from 2 to 1000) and max depth (from 3 to 18, experimentally determined range, no decrease in the BIC score was observed above 15). To save computation time, we limit the grid search for longer sequences using the observed patterns for shorter sequences. The optimal max depth as determined by BIC for each sequence ranges from 4 to 15, and correlates with sequence size (Fig. 6). However, for the min count parameter, there is no clear correlation with sequence size. To highlight this, we measure the frequency of the 5% least common *k*-mers for the optimal parameters, which ranges from 400 to 5000. For shorter sequences, this frequency exceeds all tested min count values, thus limiting the effect of the min count parameter. In contrast, for sequences with larger optimal max depths, the min count setting is important, but is almost always larger than 200, with a single exception where it is 30.

For the benchmarks, the min count and max depth are set per sequence as determined by BIC (Table 2). The literature [16, 23, 32] suggest restraining the included contexts based on their frequency, either through a min count or the relative frequency, as standard procedure to construct models capable of generalization. Our experiments with BIC further support that a comparatively large choice for min count guard against overfitting. While Cunial does not support min count, it is possible to achieve a similar effect using the four-threshold estimator [32] implemented in Cunial, but not in combination with Kullback-Leibler. Nevertheless, changing this parameter does not seem to impact the running time. Following [16, 37], we use a Kullback-Leibler threshold of 3.9075, which is half of the value of the $\chi ^2$-distribution at $3=|\Sigma | - 1$ degrees of freedom and $p=0.05$. This value has been found to be close to optimal using the AIC (a method similar to BIC) by Mächler and Bühlmann [37], and the setting was also experimentally verified here. We observed large deviations from this threshold value in combination with min count and max depth gives worse BIC scores.

**Table 2:** Optimal parameter settings as evaluated by BIC

Organism	Min count	Max depth
*Pandoravirus salinus*	*5	4
*Sorangium cellulosum*	300	6
*Drosophila melanogaster*	*2	7
*Oryza sativa*	400	12
*Symbiodinium kawagutii*	1000	13
*Homo sapiens*	800	15
*Palaemon carinicauda*	1000	15
*Pinus taeda*	1000	15

The * denotes where the min count parameter setting does not impact the optimum, as all *k*-mers are more frequent than 1000

**Fig. 7:**
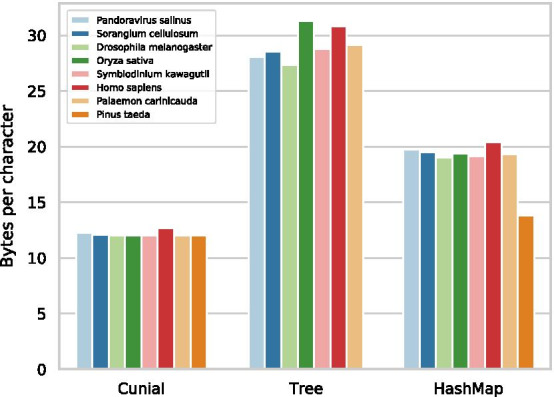
Memory per input character for the construction of each algorithm. Results for the *Pinus taeda* are missing for Tree due to insufficient memory on the benchmark server. Overall, Cunial is superior to the other approaches

**Fig. 8:**
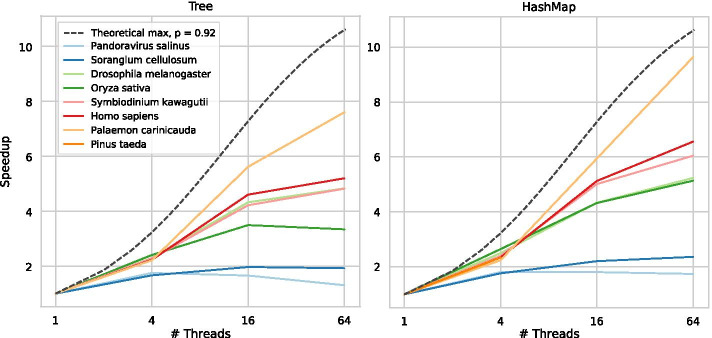
The parallel speedup of the Tree and HashMap algorithms. The speedup is compared with the theoretically optimal speedup for $p=0.92$ given by Amdahl’s law. By profiling, we measured that approximately 8% of the code is serial and cannot be accelerated, although this number differs somewhat depending on sequence size and parameters. For short sequences the speedup is small, but longer sequences have close to optimal speedup. The benchmarking machine has 32 cores, and thus the result for 64 threads illustrates that there are some benefits to scheduling more threads than cores. The HashMap exhibits better parallelisation speedup overall

During construction, both Tree and HashMap are faster than Cunial (Table 3), but are not as memory efficient (Fig. 8). Moreover, HashMap is faster than Tree and requires less memory. Compared to Cunial, the sequential Tree is between 3 and 25 times faster, and with 64 threads between 36 and 97 times faster (Fig. 7). The sequential HashMap is between 4 and 34 times faster than Cunial, and with 64 threads, between 50 and 107 times faster. We observe the smallest speedups for the longest sequences. For *Pinus taeda*, the memory usage surpasses the available memory on the benchmarking machine for Tree (Fig. 8). Furthermore, due to a small memory-overhead for more threads for HashMap, it exceeds the available memory for 16 and 64 threads. However, it likely follows the same speedup patterns as the other sequences.

**Table 3:** Running time in seconds for Cunial, Tree and Hashmap. We denote the number of threads used for every algorithm in the header

	Cunial	Tree				HashMap			
Organism/#Threads	1	1	4	16	64	1	4	16	64
*Pandoravirus salinus*	6	0.4	0.1	0.2	0.2	0.2	0.1	0.1	0.1
*Sorangium cellulosum*	28	1.1	0.6	0.5	0.5	0.9	0.5	0.4	0.4
*Drosophila melanogaster*	452	24.8	9.8	5.2	4.7	20.8	8.8	5.1	4.2
*Oryza sativa*	1396	159	45	31	33	96	36	22	18
*Symbiodinium kawagutii*	3472	451	98	53	46	253	93	44	37
*Homo sapiens*	13431	1280	574	278	246	1125	438	200	156
*Palaemon carinicauda*	24624	4188	1893	746	551	4807	1880	709	437
*Pinus taeda*	82756	*	*	*	*	20804	8895	*	*

The * denotes cases where the algorithms take more memory than available on the benchmark machine

During scoring of sequences, the memory usage of the HashMap version is vastly superior to the Tree algorithm. The Tree requires the same data structures in memory as during construction, in addition to the sequence that will be scored. Thus, it takes $\ge 28$ bytes per character during scoring, slightly more than during construction. However, the HashMap only needs a hash-map in memory, which uses $\le 1$ bytes per character, much smaller than the other data structures used during construction, but dependent on the parameters. In total, scoring a sequence with the HashMap algorithm uses about bytes per character. This is comparable to the Cunial algorithm, which also uses $\le 3$ bytes per character [24]. The runtime of scoring sequences is the only case where the Tree outperforms the HashMap. For example, scoring of a sequence with 50 000 characters takes 10ms for the HashMap, but only 4ms with the Tree. This is between 20 and 100 times faster than the Cunial algorithm on a sequential run. Similar speeds per character are observed on long genomes (results not shown). Furthermore, we implement basic parallelisation by either splitting a sequence and running each piece in parallel, or by running many sequences in parallel for a linear speedup in scoring time.

**Fig. 9:**
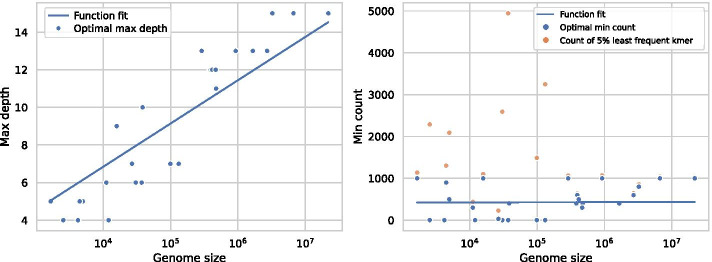
The optimal parameter settings estimated using Bayesian information criterion. The max depth correlates with sequence size (spearman correlation of 0.94). In contrast, the min count parameter does not correlate with sequence size (spearman correlation of 0.25), and every *k*-mer in the tree at the optimal depth occurs more frequently than any parameter we test. Therefore, we also include the frequency of the 5% least frequent *k*-mer in the tree. The x axis is log-scaled. The function fit is a logarithmic function of the sequence size $y=c + \log (ax)$, with a standard error of 1.67 for the max depth

The parallelisation speedup during construction of the HashMap is slightly better than the Tree (Fig. 9). For short sequences, the speedup is small. However, for longer sequences and 64 threads, the Tree and HashMap achieve a speedup factor of 7.6 and 9.6, respectively. Note that the benchmarking computer only has 32 available cores, so a larger speedup factor might be possible. These results are compared to a theoretical optimum, calculated based on Amdahl’s law [44]. This law accounts for the fact that not every part of an algorithm is parallelisable. Therefore, adding more threads won’t result in a linear speedup. We experimentally determined the parallelised parts of the algorithms to constitute about 92% of the sequential runtime, although this varies depending on sequence length and parameter settings. Our algorithms do not reach the theoretical maximum, but for the longer sequences, the speedup grows roughly at the same speed as the theoretical maximum, up to 16 threads. For 64 threads, the speedup is further from optimal for all sequences but the *Palaemon carinicauda*. Notably, the difference between the theoretical optimum and the observed speedup is smaller for 4 and 16 threads compared to 64 threads (e.g. from $74\%$ of optimum to $66\%$). The imbalance in workloads for the different threads due to the GC-content further impacts the speedup, as described for the Tree algorithm in a thesis report [35].

**Fig. 10:**
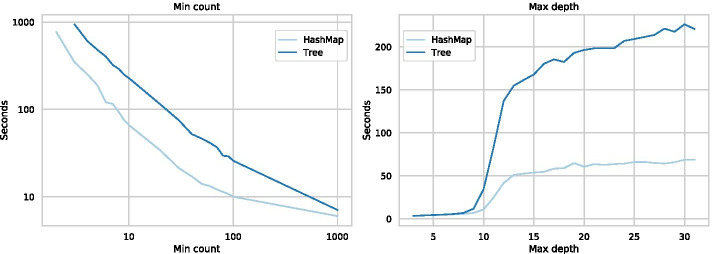
Illustration of how the parameters min count and max depth influences the runtime of the algorithms. For the min count tests, both x and y-axes are log scaled, and the max depth is set to 31. For the max depth tests, the min count is set to 10. The parameters are set to include more *k*-mers than the other benchmarks to emphasise the impact of the individual parameters. All results are run with 16 threads on *Drosophila melanogaster*. For both parameters, there are portions of exponential growth in run time, and slower growth, related to the number of included *k*-mers. The results are missing for very small min counts due to insufficient memory

**Fig. 11:**
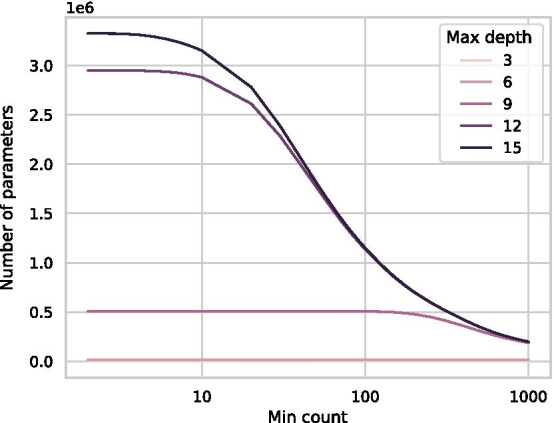
The growth of the number of included parameters at various max depths for *Drosophila melanogaster*. The x-axis is log-scaled. The number of terminal 15 and 12-mers in the model grows exponentially with min count. For smaller max depths, the min count parameter does not influence the number of parameters beyond a certain threshold, or at all. The growth decreases as the min count approaches 1 since many 15-mers (and shorter) are more common than the min-count, so the parameter does not influence the inclusion criteria as much

The values of min count and max depth have a large impact on the runtime of the algorithms (Fig. 10), related to the number of *k*-mers in the trees. Specifically, the number of *k*-mers grows exponentially with a decrease in min count, for sufficiently large max depths (Fig. 11, run on the *Drosophila melanogaster* genome). However, since most 15-mers in this sequence occur more than once, the parameter growth declines as the min count approaches 1. Also related to the number of parameters, the runtime for the Tree and HashMap grows exponentially for max depth values between 8 and 15. Similarly, as the min count decreases, the run time initially grows slowly, but past a min count threshold, the run time grows exponentially. The HashMap behaves better than the Tree for both parameters but experiences the same general behaviour.

Furthermore, we compare the fraction of cache misses of the algorithms and find the Cunial algorithm to be vastly superior in this regard (Table 4). For a min count of 10 and above, the HashMap algorithm has fewer cache misses than the Tree, which partly explains its superior performance.

**Table 4:** Percent of cache misses of the algorithms run on *Pandoravirus salinus*

Min count	Cunial	Tree	HashMap
1	3.6	50.7	62.9
10	–	32.0	19.5
100	–	15.7	5.5

Data collected using *perf* version 5.10.g2c85ebc57b3e counting cache misses and cache references. Cunial is superior to our algorithms, and HashMap is worse than Tree for a min count of 1, but better in the other cases

However, the largest cause of speedup and memory savings for the HashMap compared to Tree is that the HashMap does not need to compute the suffix links. The suffix links computation takes roughly half of the memory and a significant part of the runtime of the Tree algorithm. In the HashMap, the suffix links can instead be found with a hash lookup. Thus, traversing the tree in the HashMap is slightly slower, as evident in the runtime of scoring sequences, as it requires hashing instead of following a pointer, but it is much faster to compute than the Tree.

## Discussion

We find that constructing the full variable-length Markov chain with the lazy suffix tree is slower than computing *k*-mers with the lazy suffix tree and storing them in a hash-map.

Our BIC results give optimal max depths that are larger than those found to be optimal with BIC for higher-order Markov models [45]. These differences probably stem from a combination of the similarity pruning and the min count parameter, which are not available for higher-order Markov models. However, it is clear that these parameters have to be selected on a sequence basis, as the optimal parameter settings sometimes differ drastically between sequences.

Our parallel iteration strategy creates a thread for every shallow node, responsible for iterating its corresponding subtree. This strategy comes with the drawback that the optimal number of threads is $\Sigma ^d$ threads, with *d* as the *parallel_depth* parameter, for optimal CPU utilisation. However, it is common for computers not to have 4, 16 or 64 cores, leading to either over-subscription or under-utilisation of the available cores. Nonetheless, we have illustrated that some over-subscription can be beneficial. Moreover due to the GC-content bias of genomic sequences, it can be beneficial to create more threads than cores, to make up for the imbalance in workloads.

For short sequences, we don’t observe a large parallel speedup. In these cases, it is likely more efficient to construct variable-length Markov chains in parallel, instead of parallelising each construction.

As more unique *k*-mers are added to the lazy suffix tree, either due to the sequence length increase or inclusion criteria, the lazy suffix tree’s performance degrades. The lazy suffix tree has a worst-case running time of $O(n^2)$ [26], which is attained on highly repetitive strings.

We could reduce our memory footprint by storing the genomic sequence in a bit-compressed vector. This is possible since the DNA alphabet only consists of 4 unique values, which can be stored in 2 bits, but by default, every character is stored in a full byte. However, this has a significant runtime impact, roughly slowing down the implementation by a factor of two. Furthermore, most of the memory usage is due to vectors of integers used to index the sequence, which can’t be further compressed for long sequences.

However, it is possible to reduce the memory needed for short sequences by changing the data types of the *table* and *suffixes* vectors. These vectors index the entire sequence, which for large sequences requires 64-bit integers. However, for shorter sequences, 32-bit or 16-bit integers can suffice. We have not analysed the memory savings with these approaches, but when running many constructions of variable-length Markov chains in parallel, this might be a necessary optimisation.

## Conclusions

With the min count and max depth parameters for VLMCs suggested by BIC to avoid overfitting, the proposed implementations of VLMC construction greatly improves upon the (available) state-of-the-art. The excellent scaling of the parallel implementation, which is close to optimal for very long sequences considering Amdahl’s law, allows the construction of VLMCs even for very large genomes in a short amount of time. Further improvements are possible, as it does need to iterate the entire sequence multiple times, and requires the entire sequence and an additional vector of the same size to reside in memory. An external memory algorithm in combination with fast local disks might be a viable alternative. It is noteworthy, that we observe inferior performance from the lazy suffix tree variant for very long sequences and significant amounts of unique *k*-mers.

On a more general note: we resolved the trade-off between frequent *k*-mers, which is the main sequence features considered in Markov chains and VLMCs, and unique *k*-mers used e.g. for identification of species in environmental samples using BIC. Clearly, different choices might be reasonable for specific applications. Further investigation of respective strengths and weaknesses in relation to this trade-off might, therefore, provide further insights into sequence models, and increase our understanding of evolutionary footprints in various genomes.

## Availability and requirements

- Project name: pst-classifier.
- Project home page: https://github.com/Schlieplab/PstClassifierSeqan.
- Operating system(s): Platform independent.
- Programming language: C++.
- Other requirements: C++17 compatible compiler.
- License: GNU GPLv3.
- Any restrictions to use by non-academics: None

## Data Availability

The datasets analysed during the current study are available in NCBI, using the accession ids in Table 1.

## Abbreviations

VLMC: Variable-length Markov chain
HTS: High-Throughput-Sequencing
BIC: Bayesian information criterion
